# Computational approaches to understand transcription regulation in development

**DOI:** 10.1042/BST20210145

**Published:** 2023-01-25

**Authors:** Maarten van der Sande, Siebren Frölich, Simon J. van Heeringen

**Affiliations:** Radboud University, Department of Molecular Developmental Biology, Faculty of Science, Radboud Institute for Molecular Life Sciences, 6525GA Nijmegen, The Netherlands

**Keywords:** developmental biology, functional genomics, gene expression and regulation, gene regulatory networks

## Abstract

Gene regulatory networks (GRNs) serve as useful abstractions to understand transcriptional dynamics in developmental systems. Computational prediction of GRNs has been successfully applied to genome-wide gene expression measurements with the advent of microarrays and RNA-sequencing. However, these inferred networks are inaccurate and mostly based on correlative rather than causative interactions. In this review, we highlight three approaches that significantly impact GRN inference: (1) moving from one genome-wide functional modality, gene expression, to multi-omics, (2) single cell sequencing, to measure cell type-specific signals and predict context-specific GRNs, and (3) neural networks as flexible models. Together, these experimental and computational developments have the potential to significantly impact the quality of inferred GRNs. Ultimately, accurately modeling the regulatory interactions between transcription factors and their target genes will be essential to understand the role of transcription factors in driving developmental gene expression programs and to derive testable hypotheses for validation.

## Introduction

Multicellular organisms develop from a single fertilized egg, guided by the genetic information encoded in the genome. Cell lineages diverge and form tissues and organs, based on the interplay between signaling pathways, biomechanical forces [1] and the regulation of gene expression programs [2]. While development is controlled on many levels, transcription regulation is crucial [3]. To better understand these regulatory principles in development and evolution, it is essential to construct informative models of gene regulation.

Transcription is regulated by transcription factors (TFs) within the chromatin context [4]. TFs bind the DNA either directly, mostly in a sequence-specific manner [5], or indirectly via other TFs [6]. They can recruit various other proteins, such as co-activators, RNA polymerase, chromatin remodelers and histone modifying enzymes, to remodel or stabilize the chromatin or to activate or repress transcription [7,8]. In metazoans, TFs form up to 8% of the known proteome [9,10], with DNA binding domains and affinities being highly conserved between metazoans [11–13]. They bind specific DNA motifs that are clustered in relatively short *cis*-regulatory elements (CREs) that can be categorized as promoters, enhancers and insulators [14]. The exact function of an element depends on the combination of bound transcription factors, which is influenced by motif specificity, distance between motifs and motif directionality [15–19]. Core regulatory modules and pathways involved in germ layer and axis formation are deeply conserved in metazoans [20].

A useful abstraction to study transcription regulation is a network of transcription factors and their target genes. This concept of a gene regulatory network (GRN) was introduced in 1969 by Roy Britten and Eric Davidson and later experimentally demonstrated in sea urchin embryos [21,22]. GRNs serve to predict the effect of transcription factor expression on gene transcription and to derive testable hypotheses for validation. More generally, they function to model cell type specification and differentiation in development as well as regulatory perturbations in disease. GRNs have been constructed, mostly based on experimental loss-of-function and gain-of-function studies, for a variety of developmental models. Examples include germ layer formation in echinoderms [23–25] and frogs [26–29], neural crest formation [30,31], the *Drosophila* gap gene network [32] and hematopoietic development [33–35]. However, experimental elucidation of a limited number of interactions is hard to scale. Regulatory interactions are highly context-specific [17,36] and most remain unknown [8,37].

**Figure 1.**
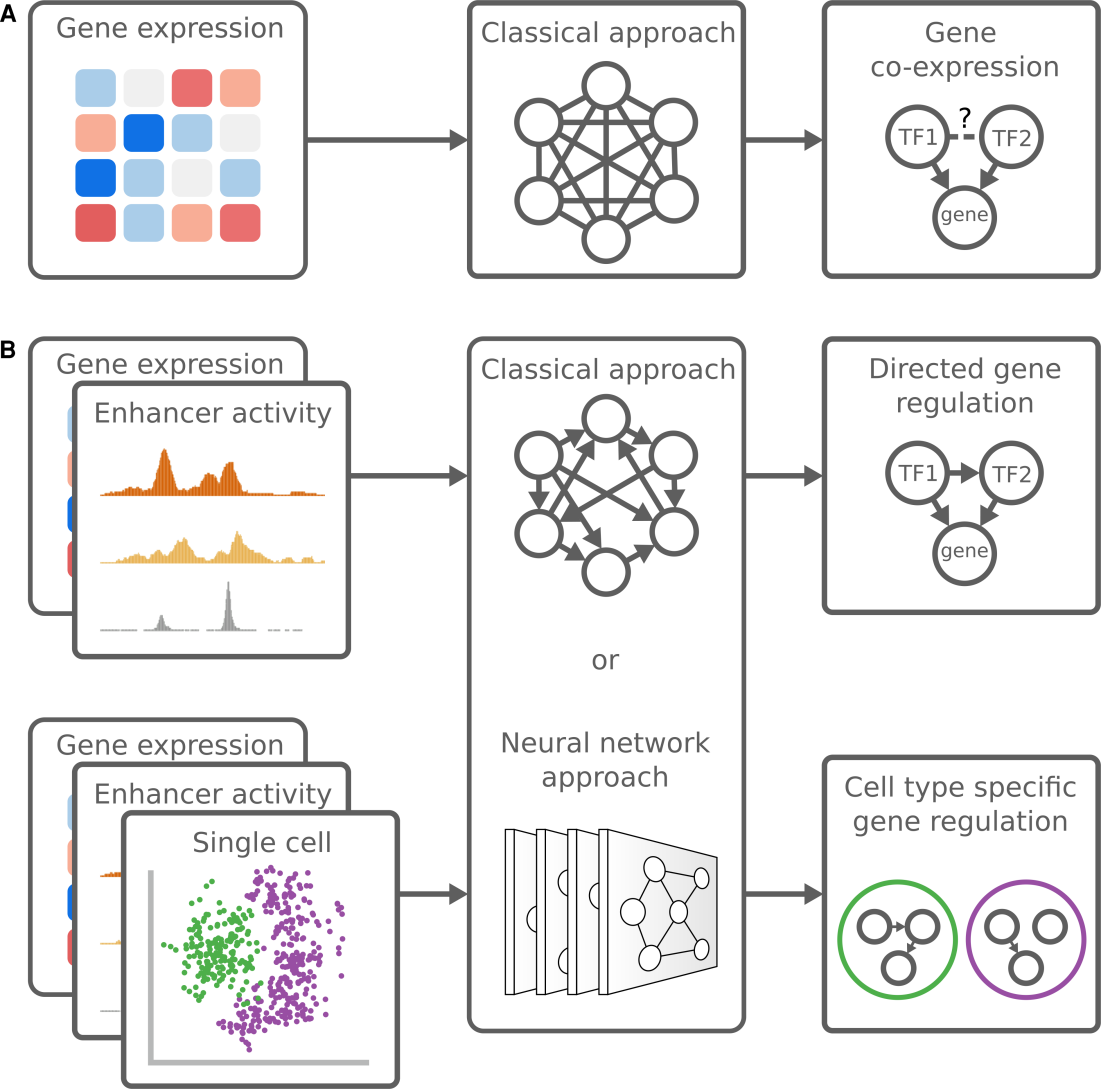
Schematic overview of different gene regulatory network inference approaches. (**A**) Classical approaches, e.g. correlation, regression or mutual information, can be applied on gene expression data to generate undirected co-expression networks. With prior knowledge about TFs the directionality between TF and target gene can be inferred, however, the directionality between two TFs cannot be established. (**B**) More recent approaches combine multiple types of genome-wide functional data (multi-omics), with either a classical approach or neural networks to identify directed gene regulatory networks. Single cell sequencing allows for the identification of cell type specific regulatory networks.

Computational inference of genome-wide GRNs was made possible with the advent of expression microarrays. Expression levels between transcription factors and their target genes tend to correlate [38] and genes with similar mRNA expression patterns are more likely to be regulated by a common transcription factor [39,40]. This led to the conception of gene co-expression networks, where functional connections between genes are inferred by expression pattern similarity. WGCNA [41] and ARACNe [42] were among the first gene co-expression-based tools and remain popular. Presently, a multitude of GRN inference methods exists. Reviews on the technical details can be found here [14,43–45]. Recent advances in experimental and computational techniques means that GRN inference has progressed beyond simple co-expression. In this review, we will highlight three approaches that have the potential to significantly impact GRN modeling: (1) moving from one modality, gene expression, to multi-omics, (2) single cell sequencing for cell type-specific signal and (3) neural networks as flexible gene regulatory models (Figure 1).

## Multi-omics to capture gene regulation

Gene regulation by TFs is mediated through CREs including promoters and enhancers. By incorporating TF binding at enhancers, regulatory networks can be constrained by direct, causal relationships. Ideally, binding of TFs would be determined experimentally with chromatin immunoprecipitation followed by sequencing (ChIP-seq) [46] or related techniques [47–49]. While large compendia of TF binding profiles in different cell types have been collected for humans [37], this effort remains unfeasible for less well-studied organisms, including most developmental model systems. With sufficient training data, TF binding can be computationally imputed [50–65], however, this does not necessarily generalize across species [66]. As a result, most current approaches use relatively simple models that combine experimentally measured CRE activity with TF binding motifs to computationally predict TF binding.

Putative CREs and their activity can be mapped genome-wide using chromatin accessibility assays, such as DNase I hypersensitive sites sequencing (DNAse-seq) [67] and Assay for Transposase-Accessible Chromatin using sequencing (ATAC-seq) [68]. The number of reads in an element can then be used as a measure for CRE activity in the experimental system [69]. ATAC-seq especially has been widely applied in developmental model systems, as it is experimentally relatively straightforward [31,70–78]. The chromatin environment can supply additional information on CRE location, function and activity. For instance, the transcriptional co-activator p300 is a histone acetyltransferase and can acytelate lysine 27 of histone H3 (H3K27ac). ChIP-seq using antibodies specific to p300 or H3K27ac can therefore identify active enhancers and promoters [79,80]. Other histone modifications that can be linked to CRE activity include H3K4me1 (enhancers) and H3K4me3 (promoters) [81].

CRE activity is determined by (in)direct binding of several TFs [82,83]. Therefore, characterizing TF binding at enhancers can identify their relative importance to the function of an enhancer. One approach to infer TF binding from genome-wide DNA accessibility is digital genomic footprinting [84], which has been used to directly infer GRNs [85,86]. However, sequence bias of the enzymes needs to be taken into account and TFs with more dynamic binding kinetics, such as some nuclear receptors, are not detected by footprint analysis [87–89]. Regardless, footprint analysis using cleavage bias correction can still be informative, especially in differential conditions [90,91]. A more routinely applied approach is to combine TF binding probabilities derived from TF motif scores with DNA accessibility. In some approaches, these are used as priors or constraints on network topology, where the network is inferred from gene expression measurements [92–94]. In alternative approaches, TF motif scores and accessibility are combined with RNA expression using regression models or co-variation of accessibility and expression [95–100].

Enhancers regulate transcription via context-dependent enhancer-promoter interactions [101], usually within a transcriptionally active domain [102]. Combined with TF binding data, these interactions allow for the inference of directed GRNs. Enhancer-promoter interactions can be identified experimentally with Chromatin Conformation Capture techniques [103–105], although this is still uncommon in non-model systems. Inferring interaction between enhancers to target genes is an active field of research. The most commonly used heuristic is to link enhancers to the nearest gene. However, this heuristic is often still incorrect [106,107]. Accuracy can be improved by combining enhancer to gene distance with TF-target gene co-expression [108]. Finally, Activity-by-Contact based models significantly outperform the nearest gene heuristic by using enhancer to gene distance and enhancer activity [109].

By combining gene expression data with at least one source of enhancer data (e.g. accessibility or interaction data), directed regulatory networks may be inferred with significantly higher accuracy compared with traditional co-expression approaches [98,110,111]. Not only does the combined approach filter out spurious interactions and add causality, but it also reduces the biases introduced by singular approaches. Therefore, we believe that the use of multiple omics will become dominant in all modalities of GRN inference approaches.

## Single cell sequencing for cell type specific regulation

Developmental transcription regulation has mainly been studied by either *in situ* hybridization [112], which maps the spatial distribution of gene expression of a small set of genes, or bulk gene expression studies [113]. The latter measures the whole transcriptome as a compound signal of all the different cells present in the sample. Single cell sequencing is a fast developing technique to measure the gene expression of individual cells separately, with newer techniques even capable of tagging cells to their spatial coordinates [114,115]. These techniques increase the number of measurements from a handful to several (tens to hundreds of) thousands. This substantial increase in data allows for interesting new ways of GRN inference, but poses new challenges as well.

The output of a single cell experiment generally consists of count tables containing several thousands of cells with low coverage, e.g. only a few thousand of measured transcripts per cell. The low coverage makes the detection of relations between lowly expressed genes difficult. Although it is possible to artificially increase the sequencing depth by simulation (imputation), this does not seem to improve GRN inference [116,117]. Furthermore, it is important to note that cells are repeated measures [118], meaning that the cells come from the same environmental and genetic background, which breaks most statistical assumptions. Computationally clustering related cells, called pseudobulk or meta-cells [119], and using their combined signal solves the issues of low coverage and repeated measures, and still yields cell-type specific signals.

Since fundamentally there are small differences between bulk and pseudobulk data, it is not uncommon to apply bulk GRN inference approaches, such as gene co-expression, ARACNE [42] and GENIE3 [120], to pseudobulk data without much adjustment.

The large number of cells, however, allows for specialized single cell GRN approaches. These include mutual information in combination with partial information decomposition [121], gene coexpression [122], self organizing maps [123], or a combination of single cell RNA-seq and single cell ATAC-seq coexpression and/or bayesian ridge regression [124–126]. Other approaches first order cells by their inferred temporal ordering and then infer the gene-gene relations on this pseudotime, with the assumption that these orderings, also called trajectories, represent cell lineages [127]. Pseudotime can be estimated by simply following the first principal component, or finding the minimal spanning tree between clusters [128], where more advanced methods smoothen the tree [129,130]. A downside of these techniques is that they can not infer the directionality of the relationships. To computationally obtain this directionality, the ratio between spliced and unspliced transcripts per gene can be used as a proxy for whether or not a gene is actively transcribed. By applying this logic across all genes and all cells, one can infer a vector field of velocities of cells which then can be used to get a temporal cell ordering with a start and end [131,132]. These orderings then allow for inferring ordinary differential equations [133,134], Granger causality [135–137], boolean networks [138] or autoregressive models [139]. Most of these methods assume Gaussian noise for gene expression, even though transcription occurs in bursts [140,141], a phenomenon that can only be captured on a single cell level. These dynamics can be modeled as a Markov process including transcriptional bursting and degradation [142]. Theoretically these mechanistic models could be great tools for hypothesis generation, but more work is needed to prove their practical usefulness. Even though the aforementioned GRN inference methods were developed for single cell data specifically, many fail to show consistent improvement over methods that were developed for bulk data, and are seemingly barely any better than purely random models [117,143,144]. Moreover, the added complexity and number of cells leads to computational scaling issues, with some methods taking several days to weeks to finish [117].

Single cell sequencing has the advantage that it disentangles the composite signal present in all biological tissues. The increased number of measurements allows for more complex GRN definitions and inference. Finally, it allows for the inference of fine grained temporal orderings necessary for GRN inference. Even though single cell GRN inference methods have not yet brought the improvements over bulk methods we hoped for, we still expect single cell GRN inference to become the new standard of the field.

## Neural networks as flexible gene regulatory models

Computational inference of a GRN depends on a lot of implicit assumptions. For example, a common assumption is that the relationship between genes is additive, which means that the effect on a gene equals the sum of the effects of two regulators separately, but in reality, gene-gene relationships are more complex and for example can include multiplicative effects [145]. A type of model that requires little explicit specification about the possible relationships in the data, but automatically learns these relationships, is an Artificial Neural Network (ANN). ANNs have been successfully applied in a variety of settings, with famously complex problems such as protein folding [146], image recognition [147], and the board game Go [148]. The successes of ANNs in these unrelated fields shows great promise for application in the field of gene regulatory inference.

Just like GRNs, ANNs consist of nodes and edges. Each edge multiplies the signal from the previous node to the next, and by applying a function to the sum of all the incoming edges the value in the next node is calculated. By adding multiple layers of nodes in between the in- and output nodes (this is where the term *deep* neural network comes from), a network is formed that is capable of learning more and more complex interactions. Learning happens by giving the model examples of input data and expected output, and based on this information the model iteratively updates (learns) its edge weights. After training, hypotheses can easily be tested by systematically querying the model for the predicted effect of certain changes. See [149] for an excellent review on the topic applied to genomics.

ANNs in genomics were first applied to predict the output of a genomic assay, for instance histone modifications in a certain cell type, by using only the DNA sequence as input. Early models showed that convolutional neural networks are capable of predicting functional effects of noncoding variants from short (10–1000 bp) genomic sequences alone [150,151]. These types of models can be used to discover composite motifs and periodic binding [15]. Additionally, these models are capable of learning complex and distal biological relations, as increasing the input sequence to 131 kb still improves accuracy [152].

Whereas ANNs in genomics have mainly been popularized on sequence data, adoption for GRN inference has been relatively slow. Different approaches consist of self-organizing maps [123], variational autoencoders [153], extreme learning machines [154], or graph convolutional neural networks [155,156]. Even though these networks differ in architectural designs, they all report higher levels of accuracy over non-ANN approaches. However, without independent benchmark studies it is hard to verify these results.

The main strength of ANNs is that they can approximate any continuous relationship in the data [157,158], with the downside that large amounts of training data are required. This makes the combination of single cell sequencing and ANNs promising, as current single cell GRN inference approaches have scaling issues [117] and ANNs train relatively fast with the use of GPUs (specialized graphics cards). Fundamentally, understanding how ANNs work is, however, much harder than understanding the classical models typically used for GRN inference. This causes ANNs to be met with skepticism and the persistent misconception that ANNs only function as a black box for predictions and its logic can not be interpreted [159]. We expect ANNs to become commonplace in the field of GRN inference due to their successes in other fields, ease of implementation with high-level programming libraries [160,161], and availability of sufficient training data due to single cell sequencing.

## Discussion

Traditional GRNs, mostly based on gene co-expression, have so far served as a useful abstraction to understand regulatory dynamics in developmental systems. However, the way GRNs are currently derived suffers from two fundamental problems. First, the classic GRN that describes TF to target gene relations remains a simplified model and, by design, cannot properly reflect the full complexity of gene regulation. In addition, they are mostly based on mRNA expression as a measure of protein expression, even though this relation is not always linear [162]. In addition, any other types of regulation between transcript and protein product, such as mRNA degradation and post-translational modification, are usually ignored. Second, experiments generally have more features (i.e. genes measured) than samples which is also known as ‘*the curse of dimensionality*'. In this underdetermined system, many different models can potentially fit to the data, and it is both practically and theoretically impossible to identify the correct model with certainty [163]. It then should not come as a surprise that benchmarks consistently demonstrate that the quality of the inferred GRNs is low [143,144,164–168]. Based on these observations it is clear that our current approach to infer GRN is not sustainable and design changes are needed. Ultimately, we expect the field to move towards GRNs inferred from neural networks trained on single cell multi-omics data.

Having said that, it is not enough to just naively apply single cell multi-omics ANNs. By adding more modalities, and making GRNs more complex, networks become even more underdetermined. This is why most multi-omics approaches use the new modalities to prune the possible TF-target gene relations, which actually reduces the degrees of freedom [98,122,125,126]. Moreover, one can use time-series data to further prune TF-target gene interactions [169], although time-series multi-omics GRN inference tools are still relatively uncommon [170–173]. In addition, computational methods such as regularization [174] and dropout [175] constrain the problem in such a way that you end up with the *simplest* fit out of likely possible fits. In addition, recent developments have made it possible to measure multiple modalities in the same cell, such as combined ATAC-seq and RNA-seq [176–178], which offers new, exciting opportunities for combining single cell sequencing with multi-omics data. ANNs, finally, have been made relatively easy to implement, can learn any type of interaction, and make no assumptions about the data (such as normality), which makes them extremely powerful GRN tools. However, it is not yet clear what the optimal architecture is for these networks, and interpreting the learned network from the ANN remains difficult.

GRN inference has become a data science, and it is time that we start treating it as such. Integrating multiple omics, several thousands of cells, and training complex machine learning models requires specialized knowledge. Common mistakes, such as treating cells from the same sample as independent [118], double dipping [179], and data leakage [180], can be avoided by proper data science training, but are unfortunately still common. Comparing the quality of GRN inference methods requires standardized benchmarks with multiple datasets, preferably a mix of experimental data and simulated data [181–183]. Simulated data has the advantage that the ground truth is known which makes benchmarking straightforward, but has the clear disadvantage that the quality of simulated data depends on its assumptions and may actually not be representative of real biological data. The DREAM challenges [164,184] and BEELINE platform [144] are great examples, with predefined datasets and quality metrics. Only by measuring network accuracy in equal settings will it be possible to properly compare methods. It is however important to note that the goal of GRN inference is to gain mechanistic insights, as opposed to getting an optimal benchmark score, which makes fair comparison between approaches hard.

All together, we expect the field of transcription regulation in development to move towards increasingly multimodal GRN inference techniques to identify causal relations between genes. Single cell sequencing adds a cell type-specific precision which bulk sequencing can not provide. Finally, we expect the adoption of artificial neural networks as the field matures in technology and formal training, as these methods are inherently more powerful as previously used techniques.

## Perspectives

- Gene regulatory networks have served as powerful models to understand gene regulatory programs in development and disease. Amongst others, these networks have been applied to model developmental patterning, to identify relevant transcription factors for cell fate transitions and to characterize deregulated transcriptional programs in disease.
- We believe three relatively recent developments will impact the computational inference of GRNs. The combination of multiple data modalities, such as RNA expression and DNA accessibility, help to constrain GRN topology and to predict directed networks. Single cell sequencing will become the *de facto* standard, as it allows for cell type-specific models and is able to provide the high number of measurements that are needed. Finally, artificial neural networks have the capability to create flexible and powerful models of gene regulation, which will benefit efficient and accurate GRN inference.
- The developments outlined above have the potential to significantly improve GRN inference. To fully exploit these approaches we have to implement common data science practices, and develop community-driven benchmarks to consistently measure the performance of different techniques.

## Abbreviations

ANN: artificial neural network
ATAC-seq: assay for Transposase-Accessible Chromatin using sequencing
CREs: *cis*-regulatory elements
DNAse-seq: DNase I hypersensitive sites sequencing
GRN: gene regulatory network
TFs: transcription factors
